# Transcriptomic Analysis Reveals Candidate Hub Genes and Putative Pathways in *Arabidopsis thaliana* Roots Responding to *Verticillium longisporum* Infection

**DOI:** 10.3390/cimb47070536

**Published:** 2025-07-10

**Authors:** Qiwei Zheng, Yangpujia Zhou, Sui Ni

**Affiliations:** School of Marine Sciences, Ningbo University, Ningbo 315211, China; 226003116@nbu.edu.cn (Q.Z.); 2311130071@nbu.edu.cn (Y.Z.)

**Keywords:** *Verticillium longisporum*, Verticillium wilt, *Arabidopsis thaliana*, root, differentially expressed genes, hub genes

## Abstract

*Verticillium longisporum*, a soil-borne fungus responsible for Verticillium wilt, primarily colonizes members of the Brassicaceae family. Using *Arabidopsis thaliana* roots as an experimental host, we systematically identify *V. longisporum*-responsive genes and pathways through comprehensive transcriptomic analysis, alongside screening of potential hub genes and evaluation of infection-associated regulatory mechanisms. The GSE62537 dataset was retrieved from the Gene Expression Omnibus database. After performing GEO2R analysis and filtering out low-quality data, 222 differentially expressed genes (DEGs) were identified, of which 184 were upregulated. Gene Ontology and Kyoto Encyclopedia of Genes and Genomes enrichment analyses were performed on these DEGs. A protein–protein interaction network was constructed using the STRING database. CytoHubba and CytoNCA plugins in Cytoscape v3.10.3 were used to analyze and evaluate this network; six hub genes and four functional gene modules were identified. The GeneMANIA database was used to construct a co-expression network for hub genes. Systematic screening of transcription factors within the 14 DEGs revealed the inclusion of the hub gene *NAC042*. Integrative bioinformatics analysis centered on *NAC042* enabled prediction of a pathogen-responsive regulatory network architecture. We report *V. longisporum*-responsive components in *Arabidopsis*, providing insights for disease resistance studies in Brassicaceae crops.

## 1. Introduction

Verticillium wilt, a soil-borne vascular disease caused by *Verticillium* spp., threatens diverse agricultural crops and ornamental plants worldwide [1]. *Verticillium* spp. primarily infect dicotyledonous plants, with common hosts including cotton, potato, tomato, eggplant, lettuce, spinach, alfalfa, strawberry, oilseed rape, sunflower, olive, and woody perennials [2,3]. Symptom presentation of Verticillium wilt varies among host plants, with no strictly uniform manifestation. Common symptoms encompass wilting, stunted growth, chlorosis, vascular discoloration, and premature senescence [2]. The genus comprises diverse ascomycete species exhibiting both sexual and asexual reproduction cycles, of which *V. dahlia* and *V. albo-atrum* are the most extensively studied [4]. As exemplified by *V. dahliae*, this fungus encodes over 700 putative secreted proteins [5]. A substantial subset of these proteins has been experimentally validated to mediate infection-related processes, including plant cell wall degradation, host immune interference, toxic metabolite secretion, oxidative stress neutralization, host nutrient competition, and host microbiota modulation [6].

While originally identified as a variant of *V. dahliae* [7], *V. longisporum* is now recognized to be a unique near-diploid hybrid species. This pathogen evolved through recurrent parasexual hybridization events involving multiple ancestral lineages, leading to its current taxonomic elevation to species rank [8,9]. Morphologically, conidia of *V. longisporum* exhibit dimensions comparable to or exceeding those of *V. dahliae* [7,8,9]. The unstable morphology of microsclerotia and variable number of sclerotial units per whorl preclude reliable differentiation of hybrid lineages through phenotypic characterization alone, necessitating molecular diagnostic approaches for precise identification [10,11]. Comparative analyses demonstrate differential host range specificity and virulence among the three fungal lineages [7,12]. The infection process parallels *V. dahliae*, commencing with melanized microsclerotia formation. Upon root exudate recognition, these microsclerotia germinate to colonize root hair surfaces before initiating tissue penetration [13,14]. In *Brassica napus*, the fungus further colonizes xylem vessels, producing conidia that induce vascular occlusion and associated pathologies [15]. Ultimately, the pathogen emerges from xylem conduits to form microsclerotia beneath the stem epidermis and within pith tissues. These dormant structures are then released into the soil through host-tissue degradation during plant senescence [16].

Investigating the molecular mechanisms of the interaction between *Verticillium longisporum* and its host plants is critical for developing effective control strategies against this pathogen. The known Verticillium wilt resistance gene, *Ve1*, was initially identified in tomato [17]; however, no authentic resistance gene has been identified specifically for *V. longisporum*. Recent genomic studies on common Brassicaceae crops provide valuable references for resistance breeding [18]. However, considering the differences in genomic and regulatory programs among various crops, and given that *V. longisporum* can still latently colonize Brassicaceae weeds posing potential threats to crops [7], it is feasible to explore more representative auxiliary targets, such as using the model organism *Arabidopsis thaliana* for research. Altered expression or deletion of certain genes has been demonstrated to reduce plant susceptibility to *V. longisporum*, including the following examples: Overexpression of *EWR1* effectively decreases Arabidopsis susceptibility to this pathogen [19], *NPF5.12* and *MLP6* confer resistance to fungal colonization through nitrate competition and participation in suberin barrier synthesis [20], loss of *CRT1a* activates the ethylene signaling pathway and enhances disease resistance [21], and *TPS23/27*, two duplicated monoterpene synthase genes, produce monoterpenes that stimulate *V. longisporum* germination and subsequent invasion [22]. Notably, certain underlying mechanisms of *V. longisporum* preclude direct extrapolation of established resistance-targeting approaches. Ulrich et al. discovered through comparison of the root transcriptome of the *Arabidopsis* coi1 mutant with those of the susceptible JA-Ile-deficient allene oxide synthase mutant and the susceptible wild-type that constitutive expression of COI1—the receptor for the canonical defense hormone JA-Ile—in roots impedes synthesis and transport of defense compounds, thereby paradoxically enhancing *V. longisporum* infection efficiency [23]. Furthermore, screening for *A. thaliana* transcription factors (TFs) via overexpression enables rapid identification of *V. longisporum*-responsive regulators, thereby facilitating discovery of novel resistance targets [24].

*V. longisporum* is an emerging global threat to Brassicaceae crops, particularly *Brassica napus*. Current Verticillium wilt research has mostly focused on *V. dahlia* and *V. albo-atrum*, with limited transcriptomic analyses available for *V. longisporum*. Using bioinformatics approaches with the susceptible model organism *A. thaliana* [7], we perform comprehensive transcriptomic profiling to identify potential hub genes and associated pathways, and elucidate molecular regulatory networks responsive to *V. longisporum* infection. Our objective is to advance disease-resistant breeding strategies and molecular-targeted interventions in Brassicaceae crops.

## 2. Materials and Methods

### 2.1. Biological Materials and Pathogen Inoculation

Wild-type *A. thaliana* Col-0 was used as the experimental host. Surface-sterilized *Arabidopsis* seeds were aseptically inoculated onto Murashige and Skoog (MS) solid medium, stratified at 4 °C for 48 h, then cultivated in growth chambers for 14 d under controlled environmental conditions: 22 °C, a 16-h light/8-h dark photoperiod, and photosynthetic photon flux density of 100–150 μmol·m^−2^·s^−1^. Control group seedlings were maintained on 1/2 MS medium, while treatment group seedlings were cultured on 1/2 MS medium supplemented with *V. longisporum* spores (200 conidia mL^−1^) for 48 h, followed by root tissue collection. (The *V. longisporum* strain HZM135335 was obtained from HZBio Microbial Conservation Co., Ltd., Wuhan, China) RNA extraction was performed using the E.Z.N.A. Plant RNA Kit (Omega Bio-tek, Inc., Norcross, GA, USA), with cDNA synthesis via HiScript III RT SuperMix (ABclonal Biotechnology Co., Ltd., Wuhan, China).

### 2.2. Raw Transcriptome Data Collection

Our GSE62537 (https://www.ncbi.nlm.nih.gov/geo/query/acc.cgi?acc=GSE62537; accessed on 6 November 2024) microarray dataset was obtained from the Gene Expression Omnibus (GEO) database [25]. As a public repository, GEO hosts vast amounts of global high-throughput sequencing and microarray data. This dataset was constructed based on the Affymetrix Arabidopsis ATH1 Genome Array (GPL198), containing control and experimental group data for A. thaliana Col-0 and ndr1-1 genotypes, with three biological replicates per group. The gene expression matrix for GSE62537 is available in Table S1.

### 2.3. Data Processing and Integration

DEGs were analyzed using the GEO2R online tool within the GEO database. GEO2R is an interactive online analytical platform designed to compare two or more sample groups in GEO datasets and identify genes with differential expression under experimental conditions. Visualize the expression profiles of these genes in the form of volcano plots. Probes mapping to multiple genes were rigorously filtered to enhance result accuracy. Selection criteria were set to *p* < 0.1 and absolute log2 fold change (|logFC|) ≥ 1. Based on fold-change analysis of these genes, we selected the top 10 significantly upregulated and downregulated DEGs, respectively. The absolute expression values were normalized via Z-score transformation, followed by heatmap visualization.

### 2.4. Enrichment Analysis of Differentially Expressed Genes

To elucidate the biological functions of DEGs, GO and KEGG pathway analyses were performed on upregulated and downregulated gene subsets. Enriched terms were filtered using a significance threshold of *p* < 0.5. To reduce redundancy, GO annotations were summarized via the REVIGO database (http://revigo.irb.hr/ accessed on 7 April 2025), with results constrained to medium similarity (0.7) and filtered by false discovery rate (FDR) values and Arabidopsis-specific annotations.

### 2.5. PPI Network Construction and Module Analysis

PPI networks were constructed by querying the STRING database (https://cn.string-db.org/, version 12.0 accessed on 7 April 2025) to investigate protein relationships [26]. Interactions with composite scores > 0.4 were considered statistically significant. The network was visualized using Cytoscape software v 3.10.3 (http://www.cytoscape.org accessed on 5 December 2024) [27]. Key functional modules were identified via the MCODE plugin in Cytoscape with parameters: K-core = 2, degree cutoff = 2, max depth = 100, and node score cutoff = 0.2. Module genes were subsequently subjected to GO and KEGG analyses.

### 2.6. Hub Gene Identification and Co-Expression Network Construction

In Cytoscape, network topology was optimized by extracting the largest connected component as the primary network via the NetworkAnalyzer tool v 4.5.0, removing isolated nodes and fragmented subnets. Six topological algorithms (MCC, MNC, Degree, Closeness, Radiality, EPC) from the cytoHubba plugin and the CytoNCA plugin were used to identify consensus hub genes. A co-expression network was then constructed using the GeneMANIA online tool (http://www.genemania.org/ accessed on 9 April 2025) to validate functional associations among these genes [28].

### 2.7. RT-qPCR Analysis

cDNA samples were prepared following the protocol described in the GSE62537 dataset. RNA samples were assessed for quality using a Nano-300 micro-volume spectrophotometer prior to reverse transcription. Primer pairs targeting hub genes were designed for quantitative real-time PCR (qPCR) analysis (Table S2). SYBR Green I fluorescent dye was used in reactions using Arabidopsis Actin2 as the internal reference. Thermal cycling conditions involved an initial denaturation at 95 °C for 3 min; 40 cycles of 95 °C for 5 s (denaturation), and 60 °C for 30 s (annealing/extension). Relative gene expression was calculated via the 2^−ΔΔCt^ method. Reactions were performed on a Roche LightCycler 480 Instrument II (F. Hoffmann-La Roche Ltd., Basel, Switzerland). SYBR Green I reagent (RK21203) was purchased from ABclonal Biotechnology Co., Ltd. (Wuhan, China).

### 2.8. Prediction of the NAC042 Regulatory Network

To investigate potential regulatory targets and molecular mechanisms underlying the response to V. longisporum, all A. thaliana TFs were retrieved from the PlantTFDB database (https://planttfdb.gao-lab.org/, version 5.0 accessed on 16 April 2025) [29]. PlantTFDB catalogs plant TFs and provides integrated analytical tools. Venn diagram analysis was performed between TFs and DEGs to identify TF-DEGs, followed by mechanistic exploration. Notably, NAC042 was identified as both a hub gene and a functional TF. As an exploratory approach, upstream miRNAs of NAC042 were predicted using the psRNATarget database (https://www.zhaolab.org/psRNATarget/seq accessed on 17 April 2025) [30], while interacting partners were mined from BioGRID (https://thebiogrid.org/ accessed on 17 April 2025) [31]. psRNATarget specializes in plant miRNA target prediction, whereas BioGRID archives experimentally validated protein–protein genetic and chemical interactions across species. A hypothetical regulatory network was constructed, representing a potential framework for understanding NAC042-mediated defense mechanisms.

## 3. Results

### 3.1. Phenotypic Evaluation in Response to V. longisporum Infection

**Figure 1:**
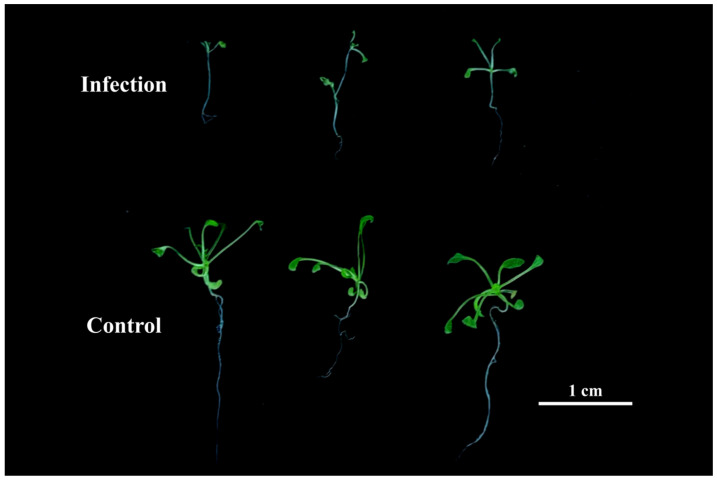
Phenotypic manifestations of *A. thaliana* under control and *V. longisporum* infection treatments at 48 h post-cultivation.

After two weeks of growth followed by 48 h vernalization, *A. thaliana* were subjected to 48 h post-inoculation treatments with either a sterile control or *V. longisporum* for systematic phenotypic evaluation. Control plants exhibited normal root and leaf development, characterized by intact primary root architecture and active lateral root formation. Infected plants had pronounced growth inhibition: rosette leaves were reduced in size, fewer in number, and wilting symptoms were visible, accompanied by suppressed root system development marked by underdeveloped architecture and significantly diminished biomass compared with controls (Figure 1).

### 3.2. Identification of DEGs Responsive to V. longisporum Infection

**Figure 2:**
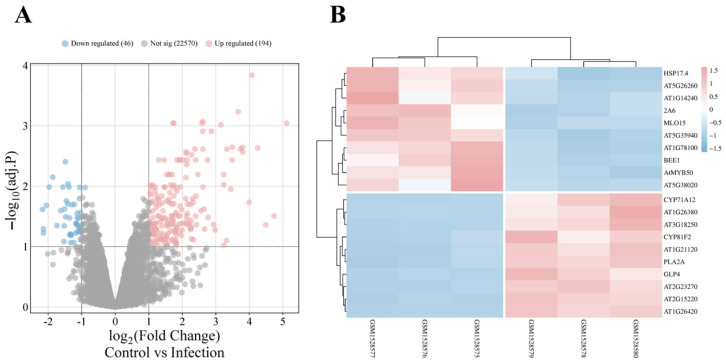
Analysis of DEGs. (**A**) Volcano plot analysis of three control versus three *V. longisporum*-infected wild-type *A. thaliana* samples from the GSE62537 dataset. (**B**) Heatmap analysis of the top 10 most significantly up- and downregulated DEGs (following stringent filtering), showing stark differential expression patterns between treatments.

To investigate *A. thaliana* roots’ responses to *V. longisporum*, 240 differentially expressed genes (DEGs) were identified between selected sample groups in GSE62837, including 194 that were upregulated (Figure 2A). To enhance quantification accuracy and experimental reproducibility, reads mapping ambiguously to multiple genomic loci were filtered out; 222 high-confidence DEGs were retained, including 184 that were upregulated. The top 10 upregulated and downregulated genes are listed in Table S3. Heatmap analysis reveals pronounced expression divergence between control and infected groups through stark red–blue color demarcation (Figure 2B). Notably, excluded multi-mapped loci may retain biological significance, as exemplified by the tandemly duplicated *TPS23/27* genes, whose monoterpene products have been implicated in promoting *V. longisporum* hyphal growth and proliferation [22].

### 3.3. Functional Characterization of DEGs

**Figure 3:**
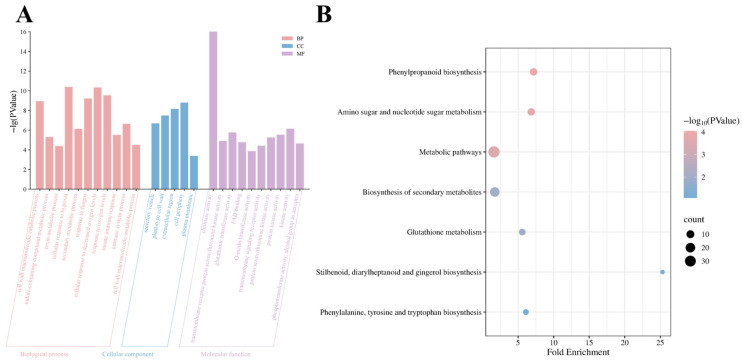
Functional and pathway analysis results of DEGs. (**A**) GO enrichment analysis. (**B**) KEGG pathway enrichment.

To investigate the roles of DEGs in *A. thaliana* responses to *V. longisporum* infection, Gene Ontology (GO) and Kyoto Encyclopedia of Genes and Genomes (KEGG) enrichment analyses were performed. Table S4 lists the most significantly enriched terms with higher enrichment scores and smaller *p*-values across categories. In the biological process category, the 184 upregulated genes were associated with cell wall macromolecule metabolic processes, indole-containing compound metabolic processes, toxin metabolic processes, cellular responses to hypoxia, secondary metabolic processes, and defense responses to fungi. For cellular components, upregulated genes were primarily linked to secretory vesicles and the dynamic interactions of the plasma membrane–cell wall continuum, including adjacent extracellular regions. Molecular function analysis revealed associations with chitinase activity, transmembrane receptor serine/threonine kinase activity, glutathione transferase activity, FAD-binding, and O-methyltransferase activity (Figure 3A). At a statistical threshold of *p* < 0.05, no significant enrichment was observed for the 38 downregulated genes. KEGG pathway analysis indicated DEG involvement in phenylpropanoid biosynthesis, amino and nucleotide sugar metabolism, biosynthesis of secondary metabolites, and glutathione metabolism (Figure 3B).

### 3.4. Construction and Module Analysis of the PPI Network

**Figure 4:**
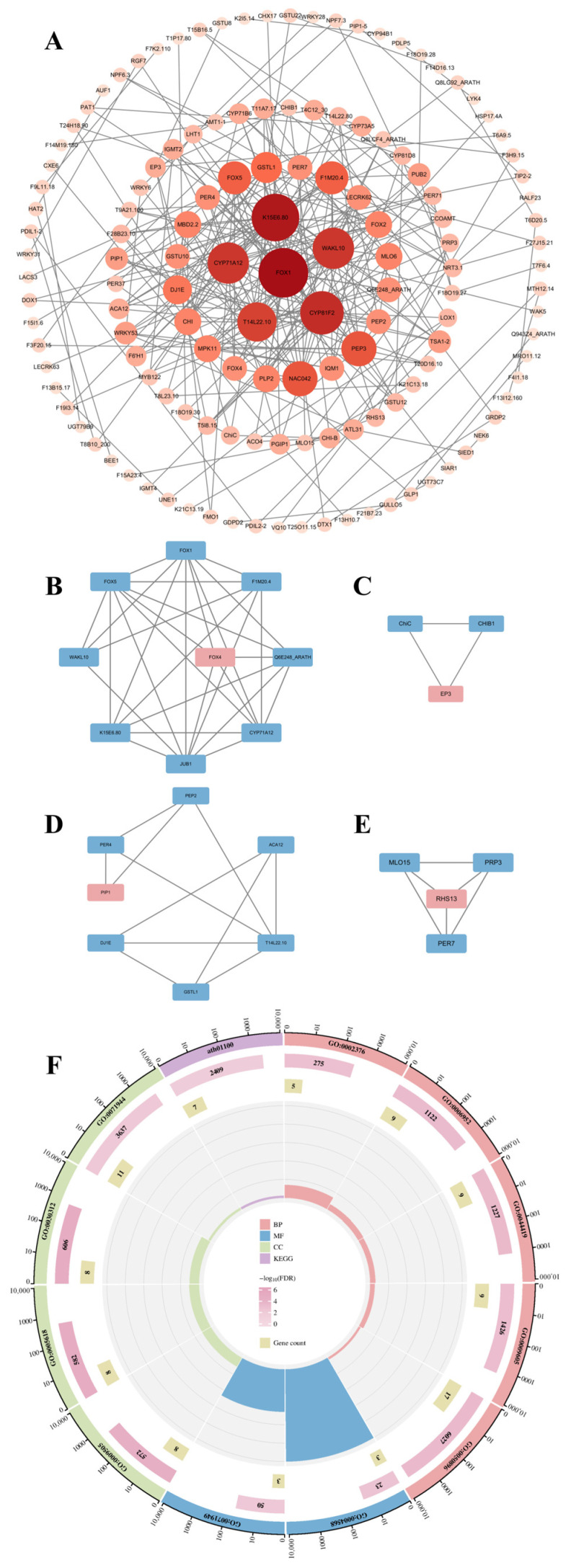
Integrated Protein–Protein Interaction Network Analysis Reveals Functional Modules and Enriched Pathways. (**A**) PPI network constructed using the STRING database. (**B**–**E**) Four gene modules identified by the MCODE plugin. Node color: red, seed genes; blue, clustered genes. (**F**) Bubble plot of GO and KEGG pathway enrichment for module-associated genes.

A protein–protein interaction (PPI) network of DEGs was constructed using the STRING database with a confidence score threshold > 0.4. The network consisted of 221 nodes and 269 interactions, with a statistically significant *p* < 1.0 × 10^−16^ (Figure 4A). The network was imported into Cytoscape for further analysis. The Molecular Complex Detection (MCODE) plugin in Cytoscape identified four tightly connected gene modules, containing 23 DEGs and 49 interactions (Figure 4B–E). GO analysis demonstrated these module-associated genes to be involved in chitinase activity, FAD-binding, immune system processes, defense response, interspecies interactions, and response to external stimuli. KEGG pathway analysis further indicated their association with metabolic pathways in *A. thaliana* (Figure 4F).

### 3.5. Comprehensive Screening and Analysis of Hub Genes

**Figure 5:**
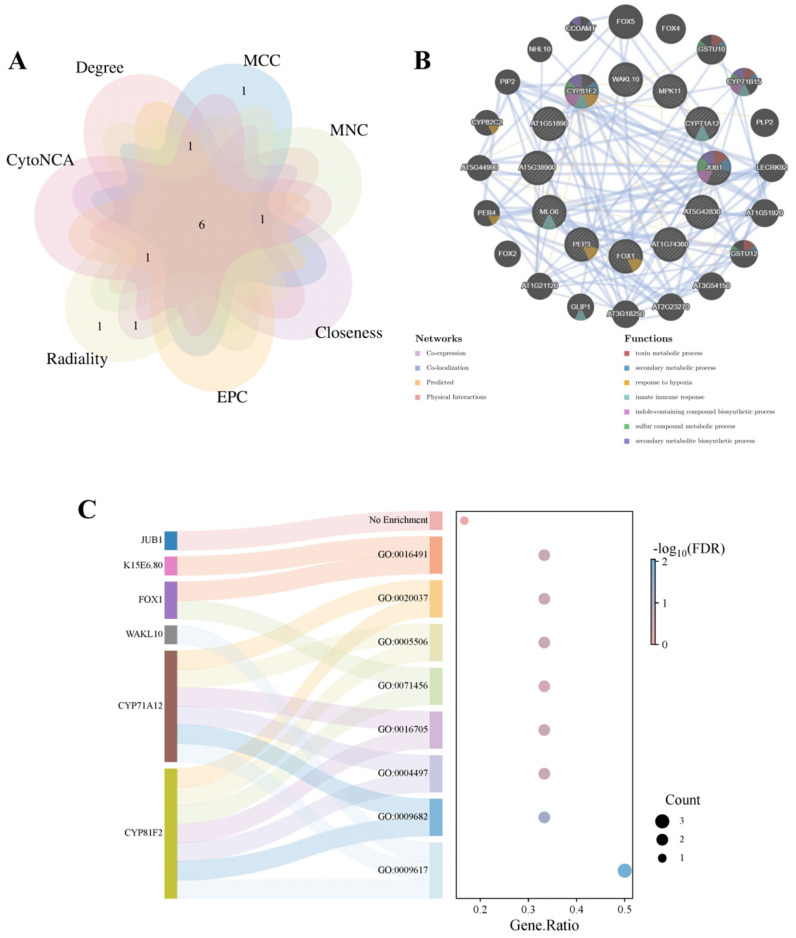
Identification of hub genes, co-expression network analysis, and functional annotation. (**A**) Venn diagram analysis of hub genes identified by cytoHubba and CytoNCA. (**B**) Co-expression network of hub genes predicted by GeneMANIA. (**C**) Mulberry bubble plot of functional annotations for hub genes; bubble size indicates gene counts and color intensity represents false discovery rate.

Hub genes were prioritized through a multi-algorithm consensus approach using six topological methods in the cytoHubba plugin (Degree, MCC, MNC, EPC, Closeness, Radiality) and CytoNCA (Network Centrality Analysis) in Cytoscape. The top 10 candidate genes from each algorithm were collated for cross-validation. To enhance screening specificity, network topology was optimized by extracting the largest connected component via NetworkAnalyzer, which removed isolated nodes and fragmented sub-networks (Table 1). Venn diagram analysis of overlapping candidates across algorithms identified six (*FOX1*, *K15E6.80*, *CYP81F2*, *CYP71A12*, *WAKL10*, and *NAC042*) high-confidence hub genes (Figure 5A). Basic information for these genes is presented in Table 2. Using the GeneMANIA database, co-expression networks and associated functions of hub genes were analyzed. These genes formed complex co-expression networks, wherein functional associations among target genes were mainly mediated by co-expression (98.79%), strongly supporting transcriptional-level functional synergy. This indicates that these genes may be coordinately regulated during *A. thaliana* responses to *V. longisporum* infection. Additionally, co-localization accounted for 1.13%, and predicted interactions for 0.08%; physical interactions fell below the detection threshold (<0.01%). These observations suggest that functional associations among hub genes likely depend on transcriptional coordination or metabolic pathway alignment rather than direct protein–protein interactions. These genes are associated with toxin metabolic processes, secondary metabolic processes, response to hypoxia, innate immune responses, indole-containing compound biosynthesis, sulfur compound metabolic processes, and secondary metabolite biosynthesis (Figure 5B).

Functional annotation of hub genes via the DAVID database identified three pathogen-response genes: *CYP81F2*, *CYP71A12*, and *WAKL10*. Of these, *CYP81F2* and *CYP71A12* were annotated to systemic acquired resistance, monooxygenase activity, oxidoreductase activity acting on paired donors, iron ion binding, and heme binding. *CYP81F2* and *FOX1* were linked to cellular response to hypoxia, and *K15E6.80* and *FOX1* were associated with oxidoreductase activity. *NAC042* received no functional annotation. The repeated mention of *CYP81F2* strongly suggests its critical role in hub gene interactions (Figure 5C).

### 3.6. Analysis of RT-qPCR

**Figure 6:**
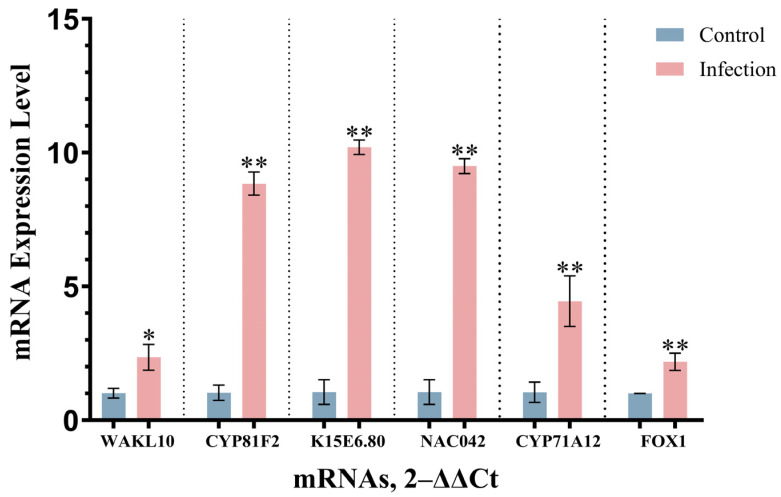
Expression of hub genes in qRT-PCR analysis. * *p* < 0.05, ** *p* < 0.01.

Quality assessment confirmed that A260/A280 and A260/A230 ratios for RNA samples from both control and experimental groups complied with established quality thresholds. Quantitative real-time polymerase chain reaction (qRT-PCR) was used to measure and compare the expression of identified hub genes between control and *V. longisporum*-infected A. thaliana (Figure 6). Expression levels of all hub genes in infected plants were significantly upregulated compared with controls (*p* < 0.05).

### 3.7. Identification of TFs in DEGs and Prediction of Upstream miRNAs

**Figure 7:**
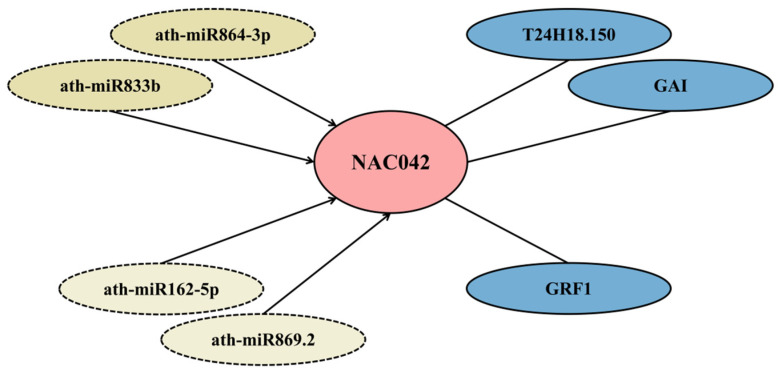
Predicted regulatory network of NAC042. Colors: dark yellow, high-confidence miRNAs; light yellow, moderate-confidence miRNAs; and blue, interacting genes/proteins. Arrows denote putative negative regulation; solid lines indicate experimentally validated interactions.

The transcription factor (TF) list of *A. thaliana* obtained from PlantTFDB comprised 2296 TFs. Intersection analysis with 184 DEGs revealed 10 to be upregulated and four to be downregulated compared with the control group, including the hub gene *NAC042*. Detailed information on all TF-DEGs is listed in Table 3. Of these, four genes originated from the WRKY family (the highest representation), and three from the MYB family; the remainder were distributed among NAC, bZIP, HD-ZIP, ERF, bHLH, and C2H2 families. The *NAC042* gene was concurrently identified as a hub gene. Integrated analysis of its combined significance across three dimensions (DEGs, TFs, and hub genes) suggests that it may be an important gene in the response of *A. thaliana* roots to *V. longisporum* infection (Figure 7). The specificity of qPCR amplification was confirmed by melt curve analysis, showing single-peak dissociation curves for all primer pairs (Figure S1).

To investigate potential regulatory mechanisms of the *NAC042* gene in the response of *A. thaliana* to *V. longisporum*, four upstream miRNAs were predicted using psRNATarget. High-confidence candidates included *ath-miR833b* and *ath-miR864-3p* (expectation = 5), with moderate-confidence candidates including *ath-miR162-5p* and *ath-miR869.2* (expectation = 4.5) potentially associated with plant–fungus interaction mechanisms. Protein interaction partners of *NAC042* were retrieved from BioGRID, including *GAI*, *T24H18.150* (validated by yeast two-hybrid assay) and *GRF1* (confirmed via affinity capture-MS). These components were integrated into a putative regulatory network (Figure 7).

## 4. Discussion

Current management strategies for Verticillium wilt are suboptimal. An integrated control approach is required. For *V. longisporum* specifically, conventional fungicides demonstrate limited efficacy as a standalone management strategy [7]. Resistant breeding is an effective approach for Verticillium wilt management. While multigenic resistance to Verticillium wilt has been identified in several crops [2,32,33], no monogenic resistance genes specifically targeting *V. longisporum* are known. Therefore, studying it through omics and modern molecular biology has more practical significance. By analyzing transcriptome data, we identified DEGs in wild-type *A. thaliana* under *V. longisporum* infection from the GEO database, including 184 that were upregulated and 38 that were downregulated. GO analysis revealed upregulated gene enrichment profiles to align with the established immune response framework in *Arabidopsis*.

Specifically, biological process enrichment related to cell wall macromolecule metabolic processes may correlate with plant responses to pathogen-induced cell wall damage [6]. Furthermore, cell wall remodeling and repair are conserved resistance mechanisms against *V. dahlia* in plants [34]. This suggests a potential conserved mechanism in defense against *V. longisporum*. Glucosinolates, a class of secondary metabolites predominantly found in Brassicaceae plants, are categorized into aliphatic, aromatic, and indolic subtypes [35]. Upregulation of glucosinolates occurs in multiple infected plant species and may contribute to resistance mechanisms against *V. longisporum* [7,36,37]. Furthermore, *V. longisporum* infection induces tryptophan biosynthesis and subsequent tryptophan-derived metabolism. Upregulated genes were enriched in indole-containing compound metabolic processes, suggesting potential crosstalk between enzymatic components regulating indolic glucosinolates, exemplified by the cytochrome P450 family gene *CYP81F2* [38]. While shared precursors such as tryptophan and its derivatives may contribute to this process, Iven et al. [39] demonstrated no significant accumulation of indolic glucosinolate breakdown products or detectable contributions from known tryptophan-derived phytoanticipins in defense against *V. longisporum*. This indicates the involvement of other unidentified indole-derived secondary metabolites in this response. In the cellular component category, the plasma membrane–cell wall continuum serves as the primary interface during early *A. thaliana*–*V. longisporum* interactions, where effector proteins and enzymes from both organisms are potentially transported via secretory vesicles [40]. Regarding molecular functions, chitinases emerge as critical extracellular hydrolases capable of degrading fungal cell wall components through β-1,4-glycosidic bond cleavage [41]. Serine/threonine kinases constitute core components of the MAPK signaling cascade [42]. The MAPK pathway regulates phenylpropanoid biosynthesis by modulating key enzymatic activities, including FAD-binding and O-methyltransferase functions, thereby driving lignin biosynthesis, energy metabolism, and phytoalexin modifications [43]. Furthermore, MAPK activation induces chitinase expression through WRKY TF-mediated transcriptional reprogramming [44]. KEGG pathway analysis revealed significant enrichment of these genes in phenylpropanoid biosynthesis, amino/nucleotide sugar metabolism, secondary metabolite biosynthesis, and glutathione metabolism pathways. Glutathione, a pivotal reducing agent, scavenges reactive oxygen species (ROS) through redox reactions and modulates defense gene expression via its metabolic flux [45,46]. Intriguingly, the MAPK cascade not only orchestrates glutathione biosynthesis and turnover but it may also be reciprocally regulated by glutathione homeostasis [47,48,49].

PPI networks enable systematic analysis of functional regulatory mechanisms, cellular signaling pathways, and disease-associated molecular targets through interaction relationship mapping [50]. We constructed a PPI network using a stringent composite score threshold (>0.4), comprising 221 nodes and 269 edges. The observed interaction count significantly exceeded random network expectations (*p* < 0.01, hypergeometric test), indicating strong functional interconnectivity among these proteins. MCODE analysis identified four densely interconnected modules with elevated node clustering coefficients (average 0.72 vs. 0.31 in random networks), containing 23 DEGs and 49 high-confidence interactions. GO and KEGG pathway analyses revealed significant enrichment of these modules in immune regulation (FDR = 2.1 × 10^−5^) and metabolic processes (FDR = 4.8 × 10^−4^), suggesting coordinated functional modules during pathogen response.

The PPI network was analyzed using cytoHubba (six topological algorithms) and CytoNCA plugins. Intersection analysis identified six high-confidence overlapping hub genes: *FOX1*, *K15E6.80*, *CYP81F2*, *CYP71A12*, *WAKL10*, and *NAC042*. Serving as highly interconnected nodes within the PPI network, hub genes are functionally pivotal to core biological processes and may represent potential therapeutic targets or biomarkers. *CYP71A12*, a putative cytochrome P450 family gene, cooperates with *CYP71A13* to biosynthesize dihydrocamalexic acid, a precursor of phytoalexins [51,52]. This gene, along with *CYP81F2*, is functionally annotated to induce systemic resistance, monooxygenase activity, oxidoreductase activity acting on paired donors, iron ion binding, and heme binding. *WAKL10* encodes a functional guanylate cyclase, although its expression is broadly induced by multiple phytopathogens, indicating limited specificity toward *Verticillium* species [53]. The *NAC042* gene, located on chromosome 2 of *A. thaliana*, functions as a gibberellin-associated TF [54]. It participates in plant defense through DELLA protein accumulation, phytoalexin biosynthesis modulation, and ROS scavenging [55,56,57]. Co-expression analysis via GeneMANIA revealed that 98.79% of functional associations among hub genes stem from co-expression, with physical interactions below detection thresholds (<0.01%). Network annotations demonstrated enrichment in toxin catabolic processes, secondary metabolic processes, hypoxia response, innate immune response, indole-containing compound biosynthesis, sulfur compound metabolism, and secondary metabolite biosynthesis. qRT-PCR validation confirmed expression patterns consistent with transcriptomic predictions.

We identified 10 significantly differentially expressed TFs through intersection of *A. thaliana* TFs from PlantTFDB with DEGs. The WRKY family contributed four genes, MYB family three genes, and bZIP, HD-ZIP, ERF, and NAC families, one gene each. As one of the largest TF families in plants, WRKY TFs regulate phytohormone signaling, MAPK cascades, ROS/calcium signaling, and phenylpropanoid metabolism to orchestrate plant immunity [58,59]. Multiple phytohormones in *A. thaliana* have been demonstrated to regulate quantitative resistance against *V. longisporum*, including salicylic acid, abscisic acid, and jasmonic acid. Additionally, this study identified that the ERECTA regulatory protein and its pathway also confer resistance to *V. longisporum*. From existing research, it is evident that the molecular regulatory mechanisms of *A. thaliana* in response to *V. longisporum* are complex and may involve further unknown potential associations [60]. All identified TF families have established roles in plant defense responses against biotic and abiotic stresses [61,62,63,64]. Notably, *NAC042* was concurrently identified as a DEG, hub gene, and functional TF, suggesting its multifunctional role in the defense of *A. thaliana* against *V. longisporum*. A *NAC042*-centered regulatory network was computationally predicted, providing insights into plant defense mechanisms against *V. longisporum* infection. Finally, all bioinformatics resources and analytical tools used in this study are comprehensively cataloged in Table S5 to facilitate experimental reproducibility and future validation studies.

## 5. Conclusions

Overall, we performed a comprehensive transcriptomic analysis of the GSE62537 dataset, including the identification of DEGs, performing GO and KEGG analyses, constructing a PPI network and identifying key functional modules and hub genes, analyzing and deriving relevant TF genes, and predicting a potential regulatory network. The TF *NAC042* was identified as a potential target for defense against *V. longisporum*. We acknowledge several research limitations. First, the analysis was based on sequencing data from public databases. Second, certain filtering criteria were applied during some analytical steps, but biological systems cannot be fully captured by simplistic selection rules (e.g., we excluded probe data corresponding to multiple genes and did not retain genes such as *TPS23/27*). Finally, because we did not experimentally validate the regulatory network, the network may contain errors or misleading elements and require quantitative analysis and functional verification.

## Figures and Tables

**Table 1:** Top 10 hub genes in cytoHubba and CytoNCA.

Degree	MCC	MNC	Closeness	Radiality	EPC	CytoNCA
*FOX1*	*FOX1*	*FOX1*	*FOX1*	*FOX1*	*T14L22.10*	*FOX1*
*FOX5*	*K15E6.80*	*CYP81F2*	*K15E6.80*	*F1M20.4*	*FOX1*	*K15E6.80*
*F1M20.4*	*CYP81F2*	*K15E6.80*	*CYP81F2*	*WAKL10*	*K15E6.80*	*CYP71A12*
*NAC042*	*CYP71A12*	*T14L22.10*	*WAKL10*	*MLO6*	*CYP81F2*	*NAC042*
*CYP71A12*	*WAKL10*	*WAKL10*	*CYP71A12*	*CYP71A12*	*CYP71A12*	*FOX5*
*WAKL10*	*T14L22.10*	*CYP71A12*	*F1M20.4*	*CYP81F2*	*WAKL10*	*T14L22.10*
*K15E6.80*	*PEP3*	*FOX5*	*T14L22.10*	*K15E6.80*	*NAC042*	*F1M20.4*
*CYP81F2*	*NAC042*	*NAC042*	*NAC042*	*NAC042*	*FOX5*	*WAKL10*
*T14L22.10*	*FOX5*	*F1M20.4*	*MLO6*	*MPK11*	*F1M20.4*	*CYP81F2*

**Table 2:** Basic information on hub genes.

TAIR ID	Gene Symbol	Full Name	Function
AT1G26380	*FOX1*	FAD-binding Berberine family protein	Within the biosynthetic pathway of the Arabidopsis cyanogenic phytoalexin 4-hydroxy indole-3-carbonyl nitrile (4-OH-ICN), *FOX1* catalyzes the dehydrogenation of indole cyanohydrin, generating indole carbonyl nitrile.
AT5G38900	*K15E6.80*	Thioredoxin superfamily protein	This enzyme exhibits protein disulfide oxidoreductase activity and participates in defense responses against fungal pathogens during incompatible interactions.
AT5G57220	*CYP81F2*	cytochrome P450, family 81, subfamily F, polypeptide 2	As a CYP81F subfamily member participating in glucosinolate metabolism, loss-of-function mutants exhibit compromised fungal resistance, while its mRNA demonstrates intercellular mobility.
AT2G30750	*CYP71A12*	cytochrome P450 family 71 polypeptide	This putative cytochrome P450 cooperates with *CYP71A13* to generate dihydrocamalexic acid (DHCA), the biosynthetic precursor of camalexin. This defense phytoalexin localizes to intercellular spaces and mediates P. syringae resistance in mature Arabidopsis plants via non-antimicrobial pathways.
AT1G79680	*WAKL10*	WALL ASSOCIATED KINASE (WAK)-LIKE 10	The encoded bifunctional kinase/guanylate cyclase protein likely participates in biotic stress response mechanisms through essential cGMP second messenger signaling.
AT2G43000	*NAC042*	NAC domain containing protein 42	This gene encodes an H_2_O_2_-inducible NAC transcription factor regulating senescence. Its overexpression significantly postpones senescence and confers enhanced tolerance to diverse abiotic stresses.

**Table 3:** Detailed Information of TF-DEGs.

TAIR ID	Gene Symbol	TF Family	Describe
Upregulated			
AT2G43000	*NAC042*	NAC	See Table 2.
AT1G62300	*WRKY6*	WRKY	Encodes a transcription factor WRKY6. Regulates Phosphate1 (Pho1) expression in response to low phosphate stress. Together with WRKY28 and WRKY41 plays a role redundant to WRKY51 in the suppression of RPW8.1.
AT4G18170	*WRKY28*	WRKY	This gene encodes WRKY6, a transcription factor that modulates Phosphate1 expression under low-Pi stress. Functionally redundant with WRKY28 and WRKY41, it cooperatively suppresses RPW8.1 alongside WRKY51.
AT4G22070	*WRKY31*	WRKY	Classified under Group II-b of the WRKY transcription factor family.
AT4G23810	*WRKY53*	WRKY	As a Group III WRKY transcription factor, it participates in antagonistic regulatory networks with WRKY53 and CRK5, collectively controlling chlorophyll metabolism, senescence progression, and stomatal conductance.
AT1G74650	*MYB31*	MYB	Member of the R2R3 factor gene family; wax regulator associated with reproductive development.
AT1G74080	*MYB122*	MYB	As an R2R3-MYB transcription factor, it modulates cuticular wax biosynthesis during reproductive morphogenesis.
AT1G06850	*bZIP52*	bZIP	This bZIP transcription factor mediates heat stress adaptation by relocating exclusively to the nucleus upon thermal challenge.
AT1G70920	*HB18*	HD-ZIP	Encodes homeodomain-leucine zipper transcription factor HD-Zip 18.
AT3G23230	*TDR1*	ERF	This gene encodes a B-3 subfamily ERF/AP2 transcription factor characterized by a single AP2 domain. The ERF B-3 clade comprises 18 members, with ATERF-1, ATERF-2, and ATERF-5 representing characterized examples.
Downregulated			
AT1G18400	*BEE1*	bHLH	Encodes the brassinosteroid signaling component BEE1 (BR-ENHANCED EXPRESSION 1). Positively modulates the shade avoidance syndrome in Arabidopsis seedlings.
AT5G47370	*HAT2*	HD-ZIP	Homeobox-leucine zipper genes induced by auxin, but not by other phytohormones. Plays opposite roles in the shoot and root tissues in regulating auxin-mediated morphogenesis.
AT3G49930	*AT3G49930*	C2H2	A zinc finger protein belonging to the C2H2/C2HC superfamily characterized by tandem zinc-binding motifs.
AT1G57560	*MYB50*	MYB	As an R2R3-MYB transcription factor, it promotes root cell elongation via transcriptional activation of PECTIN METHYLESTERASE INHIBITOR 8 (PMEI8), while its expression is suppressed by UPB1.

## Data Availability

The datasets analyzed in this study were obtained from the Gene Expression Omnibus (GEO) database (http://www.ncbi.nlm.nih.gov/geo), with accession numbers detailed in the Materials and Methods section.

## Supplementary Materials

The following supporting information can be downloaded at: https://www.mdpi.com/article/10.3390/cimb47070536/s1.
