# Supplementary figures and images for: Transcriptomic Analysis Reveals Candidate Hub Genes and Putative Pathways in Arabidopsis thaliana Roots Responding to Verticillium longisporum Infection
Source: Curr Issues Mol Biol. 2025 Jul 10;47(7):536. doi: 10.3390/cimb47070536 (PMC12293646; doi:10.3390/cimb47070536)

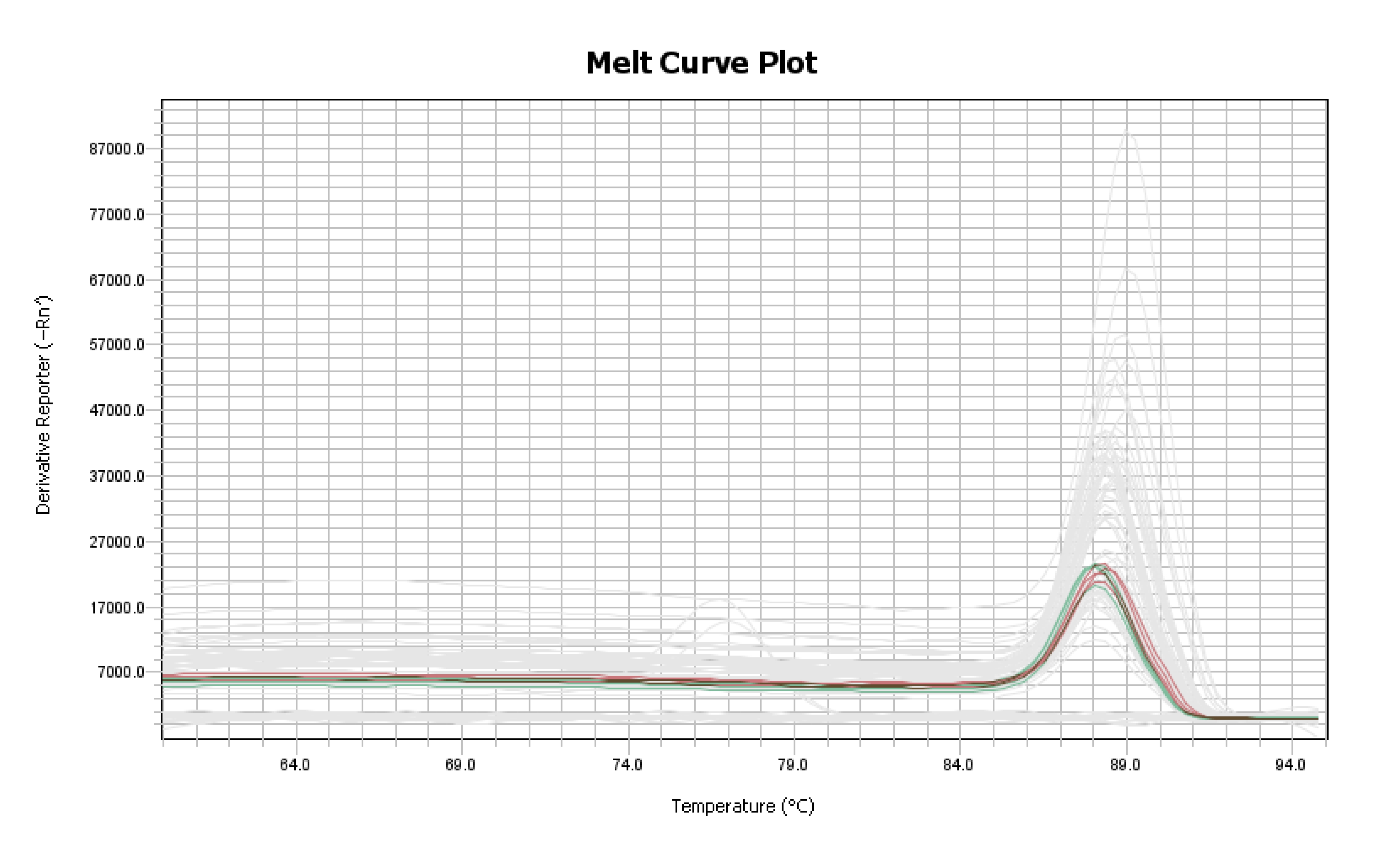

Supplement: Supplementary file 1 [file cimb-47-00536-s001.zip › Figure S1/A.jpg]

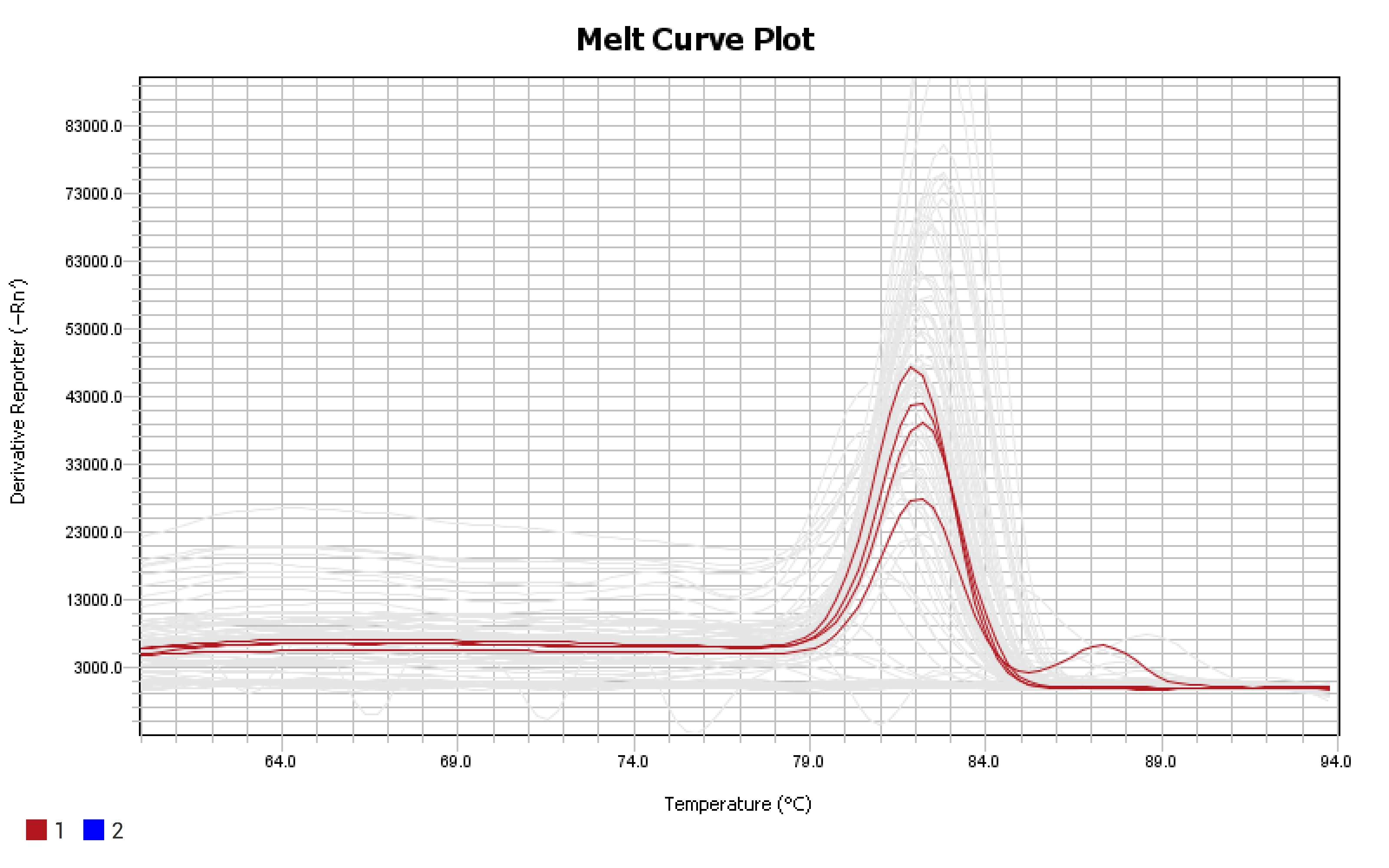

Supplement: Supplementary file 1 [file cimb-47-00536-s001.zip › Figure S1/B.jpg]

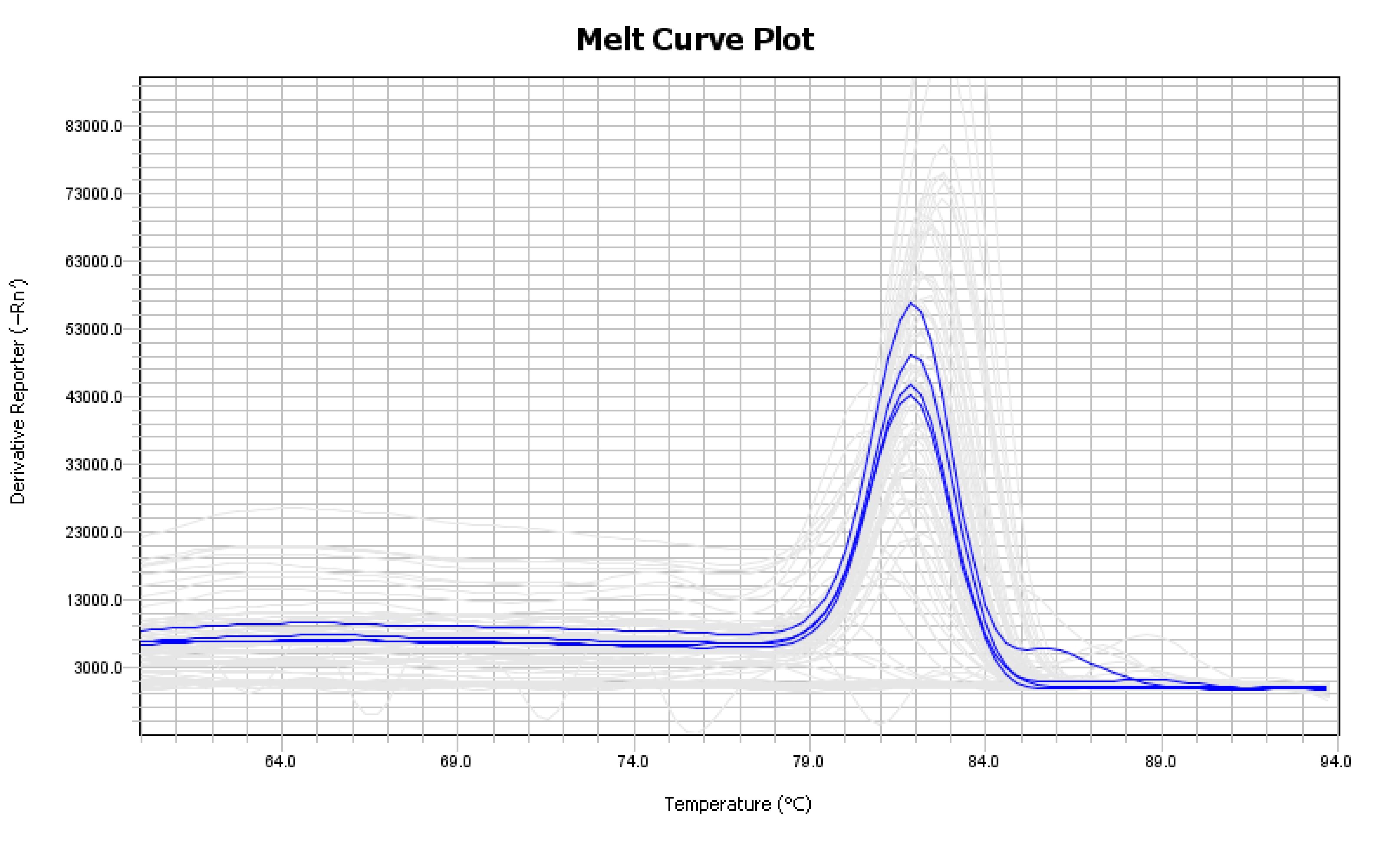

Supplement: Supplementary file 1 [file cimb-47-00536-s001.zip › Figure S1/C.jpg]

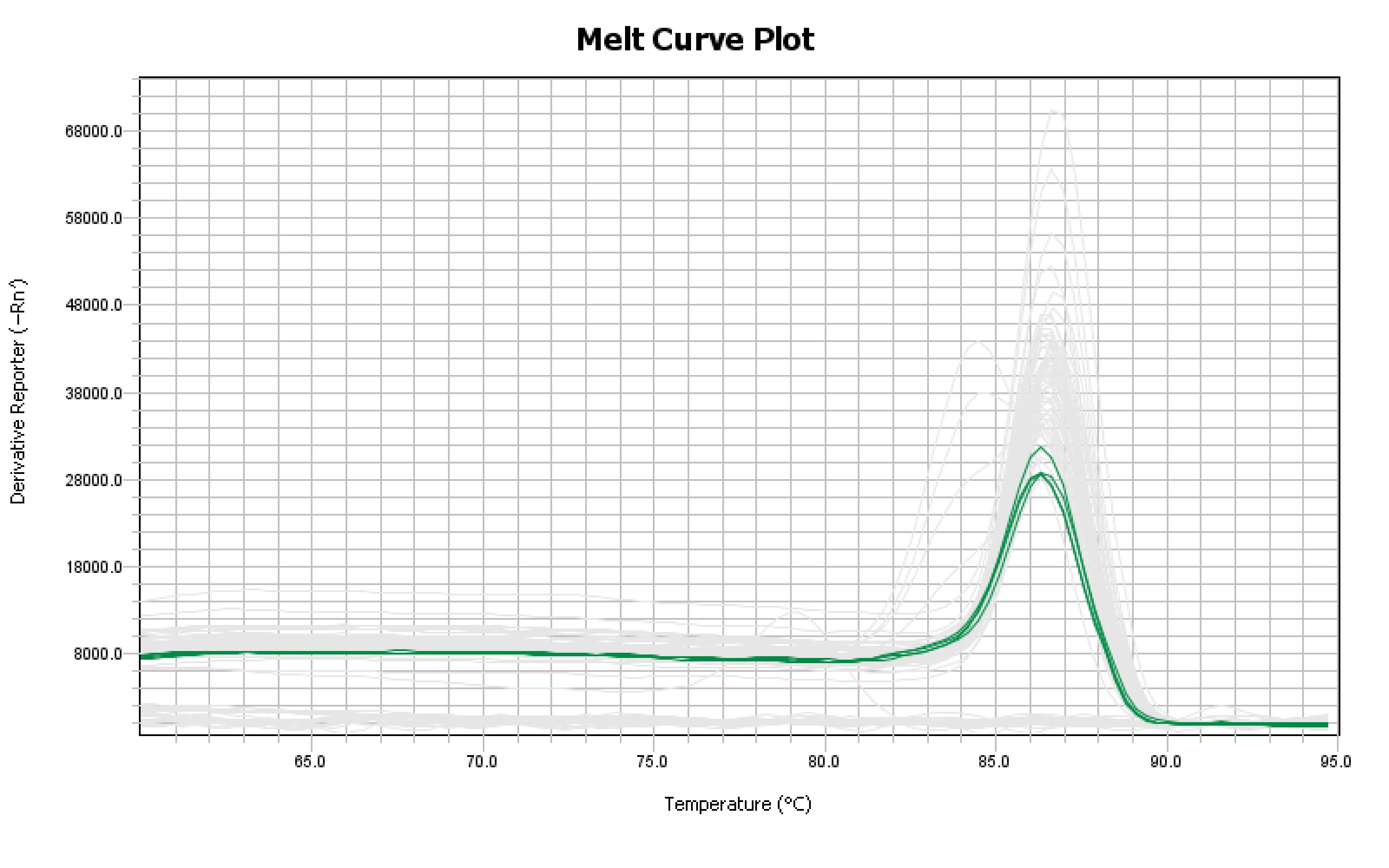

Supplement: Supplementary file 1 [file cimb-47-00536-s001.zip › Figure S1/D.jpg]

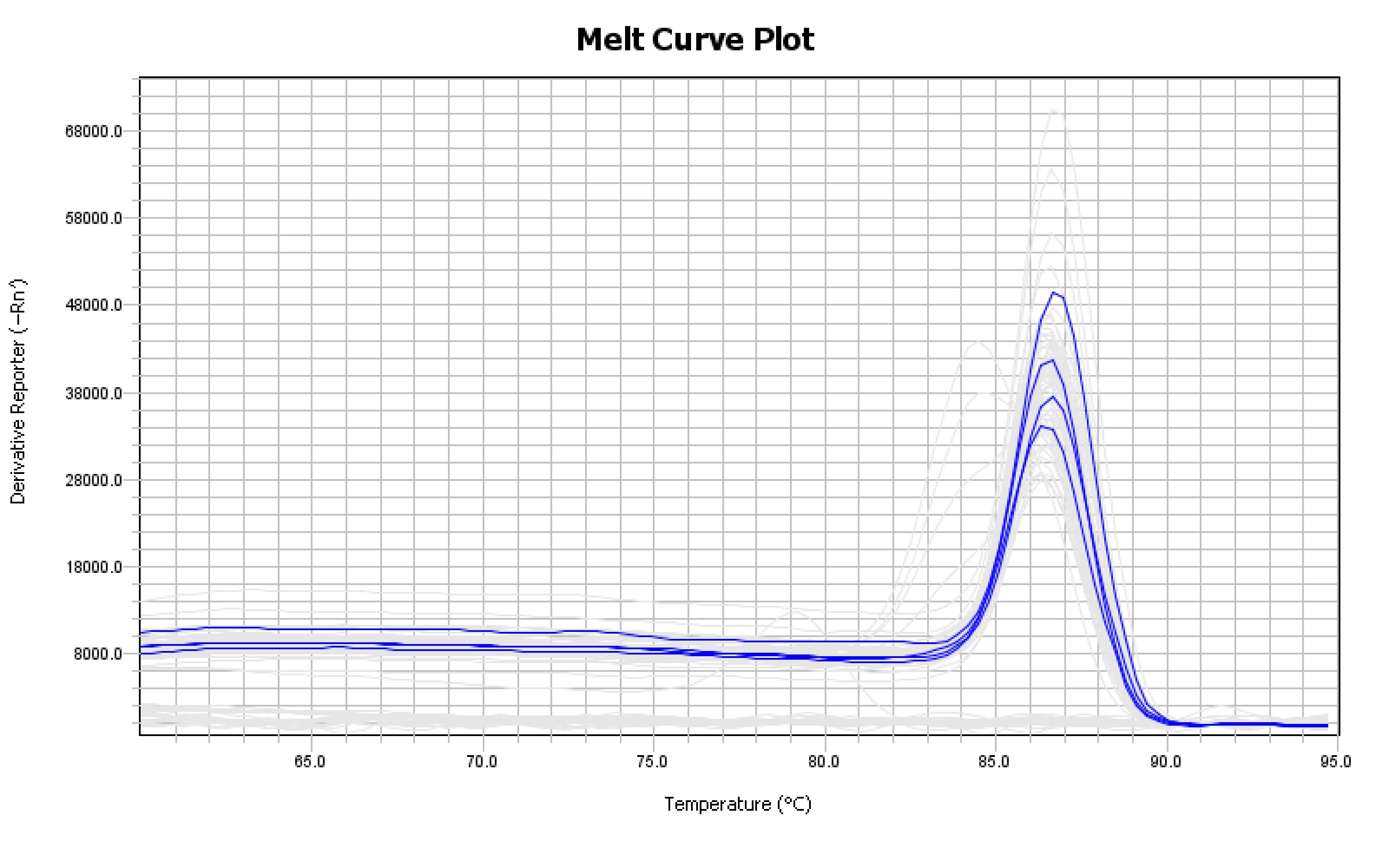

Supplement: Supplementary file 1 [file cimb-47-00536-s001.zip › Figure S1/E.jpg]

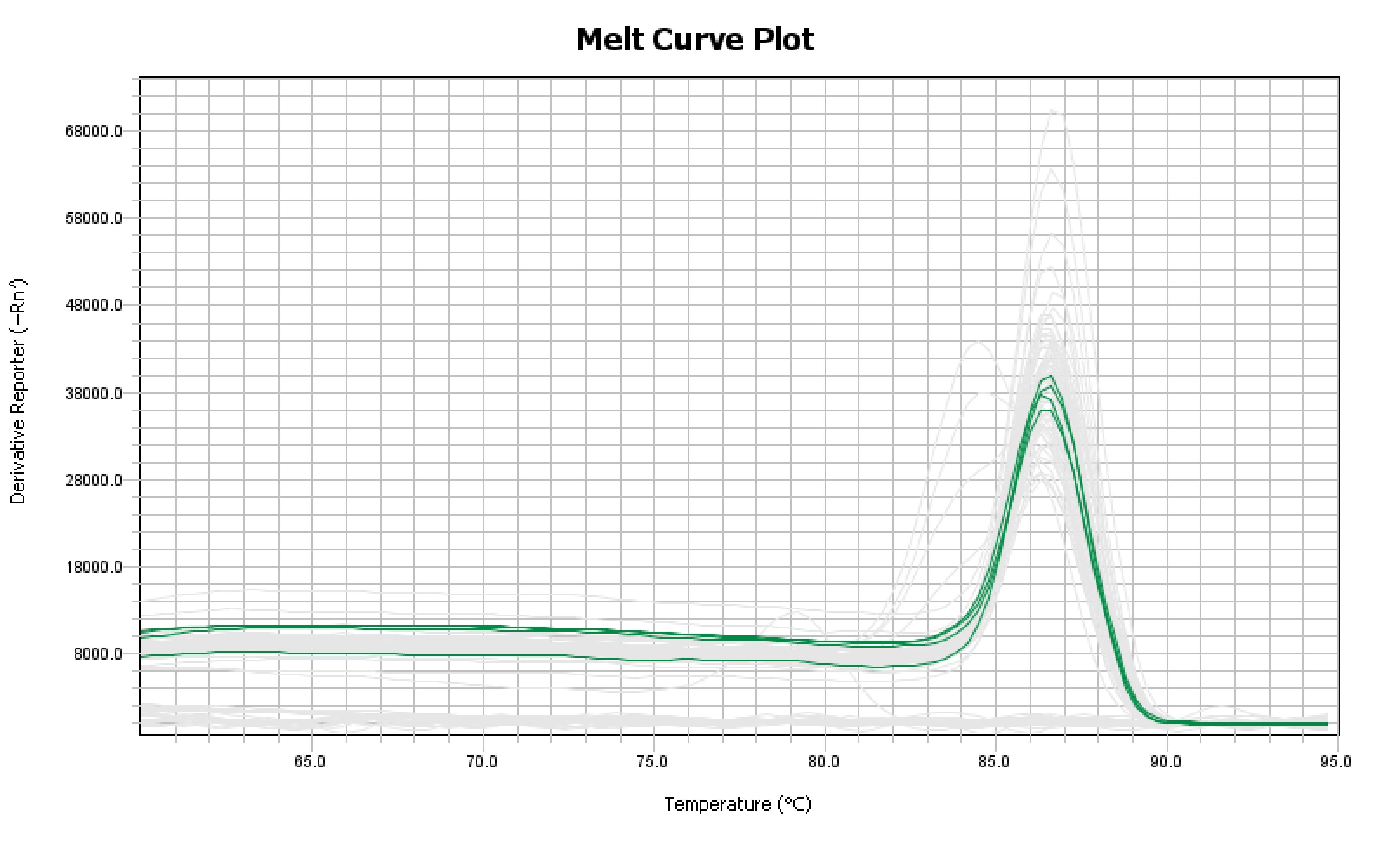

Supplement: Supplementary file 1 [file cimb-47-00536-s001.zip › Figure S1/F.jpg]

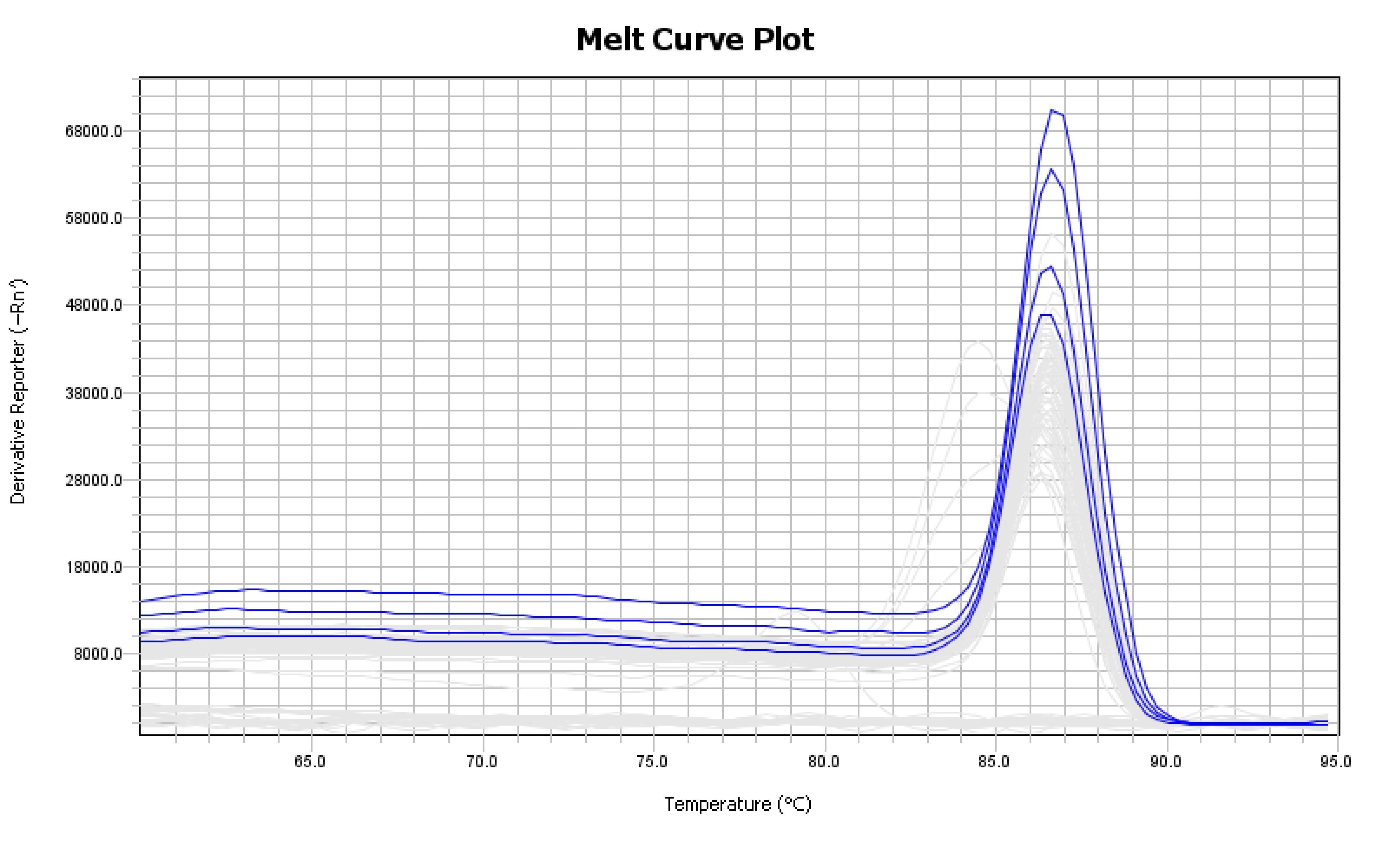

Supplement: Supplementary file 1 [file cimb-47-00536-s001.zip › Figure S1/G.jpg]

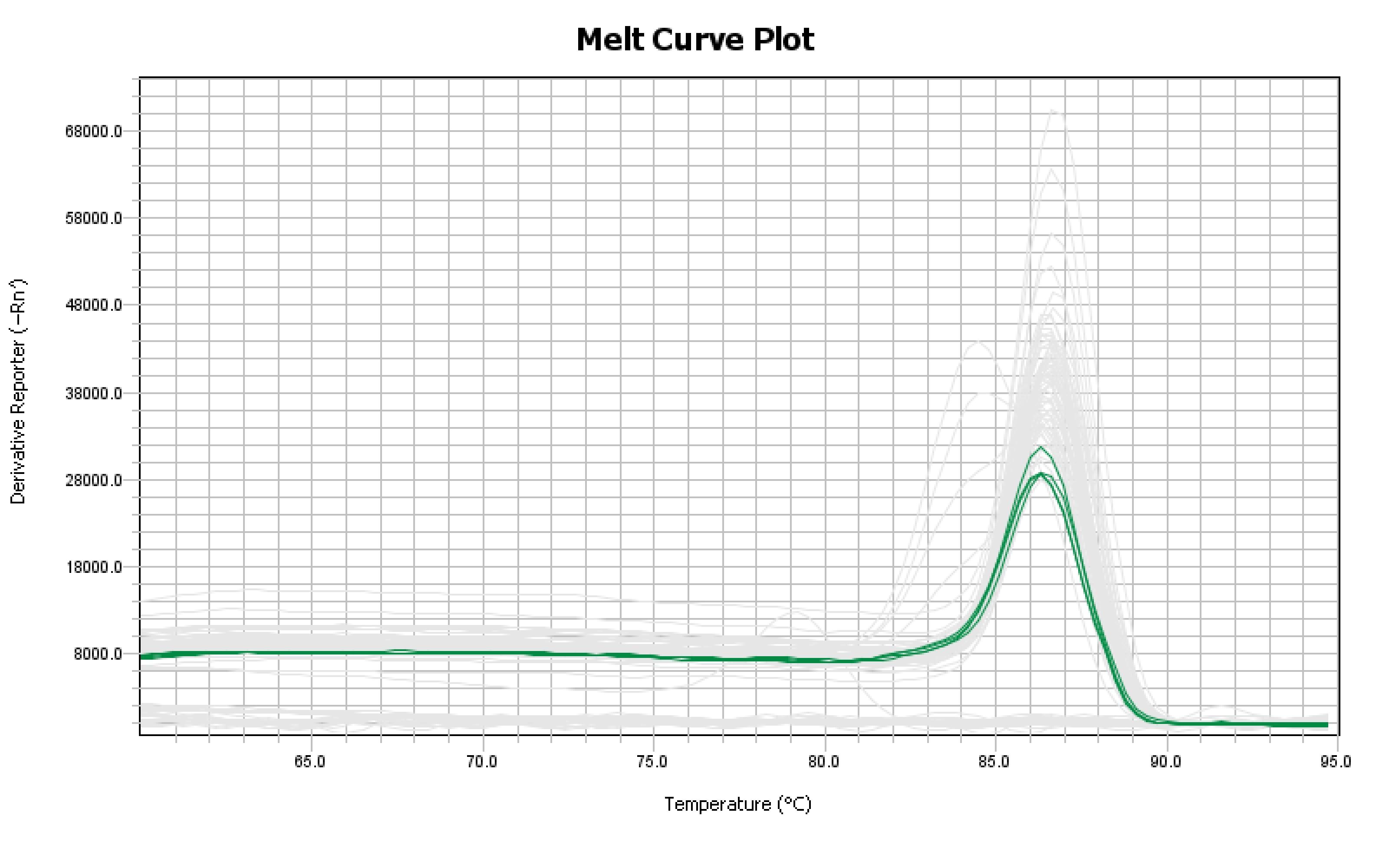

Supplement: Supplementary file 1 [file cimb-47-00536-s001.zip › Figure S1/H.jpg]

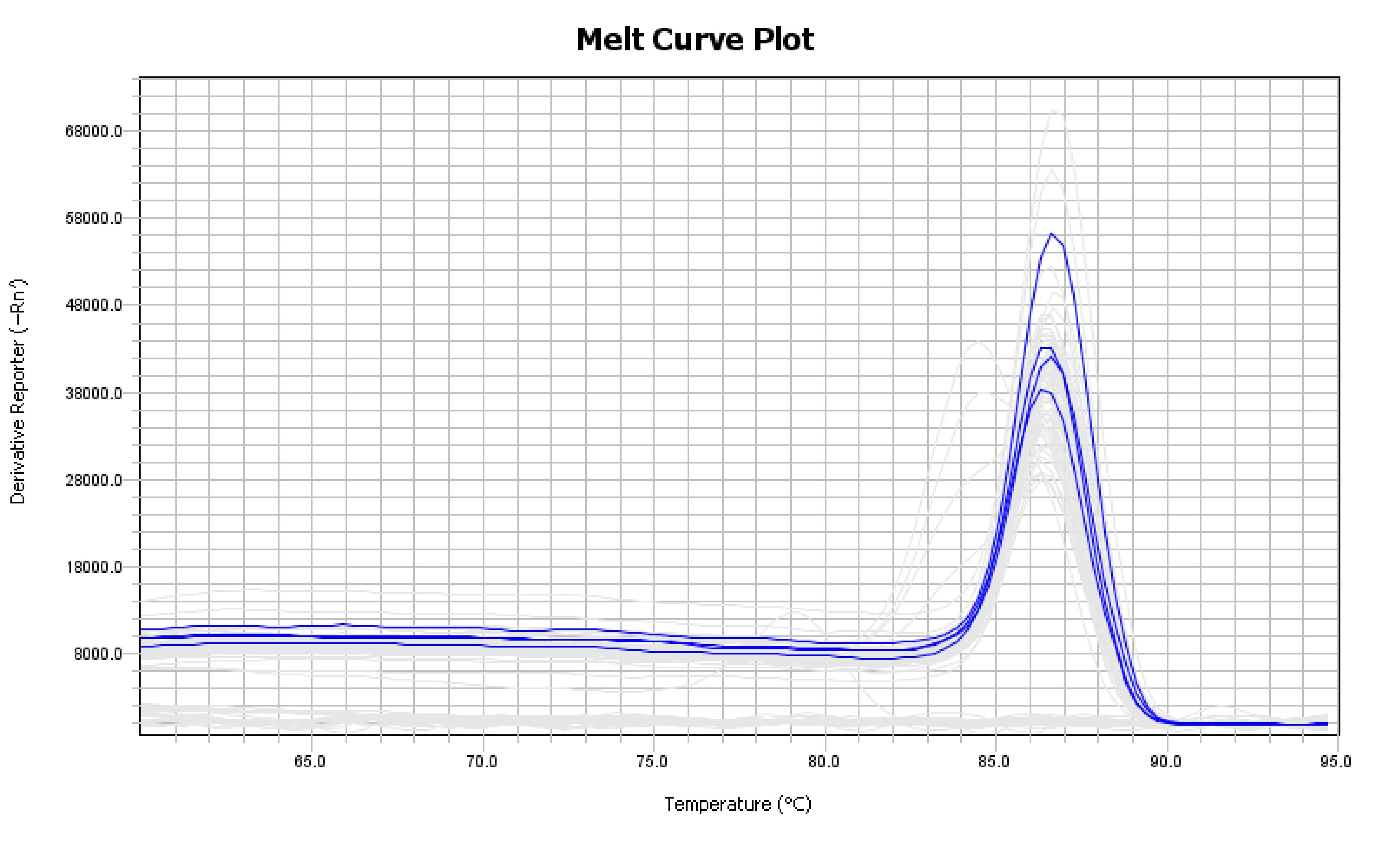

Supplement: Supplementary file 1 [file cimb-47-00536-s001.zip › Figure S1/I.jpg]

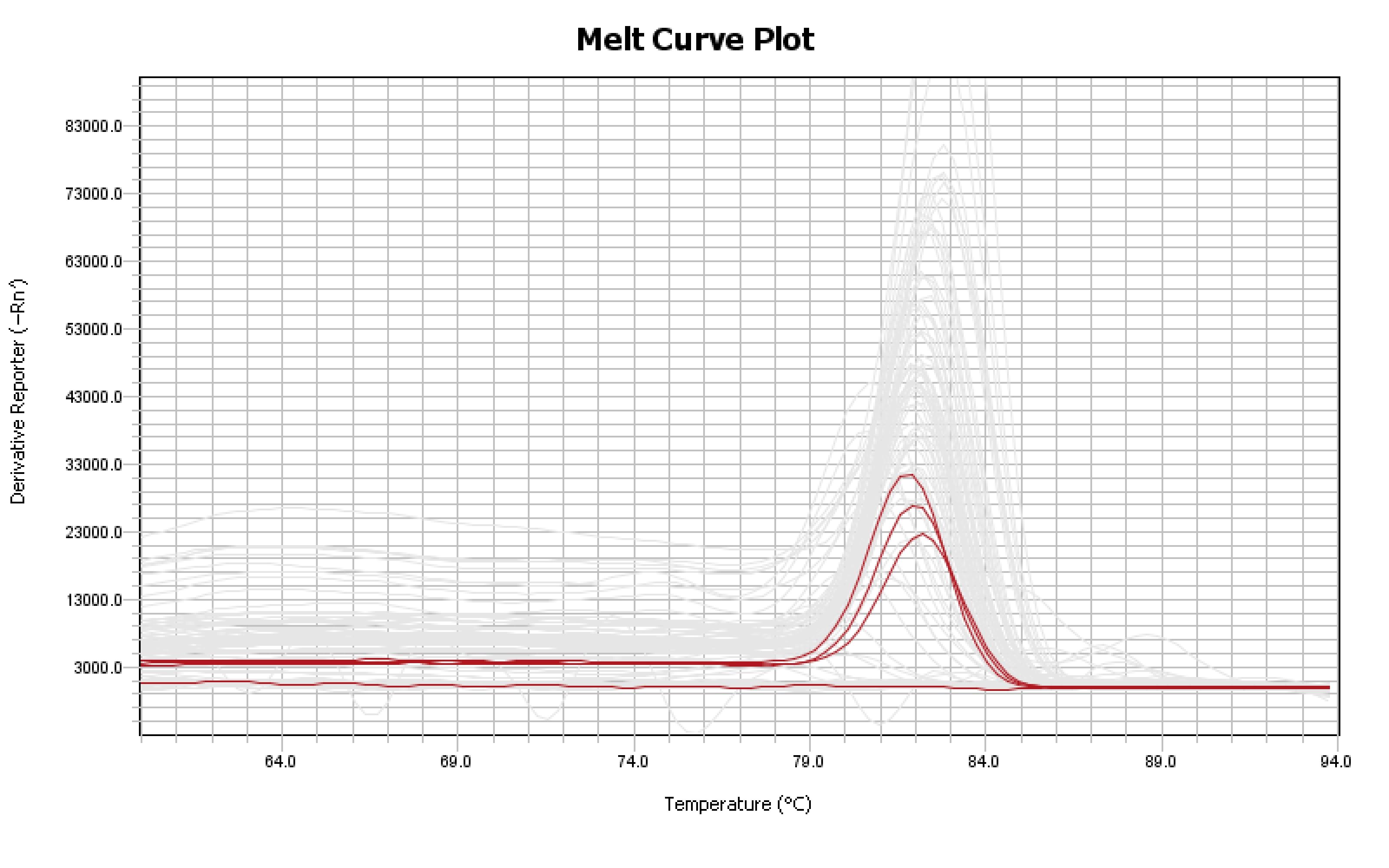

Supplement: Supplementary file 1 [file cimb-47-00536-s001.zip › Figure S1/J.jpg]

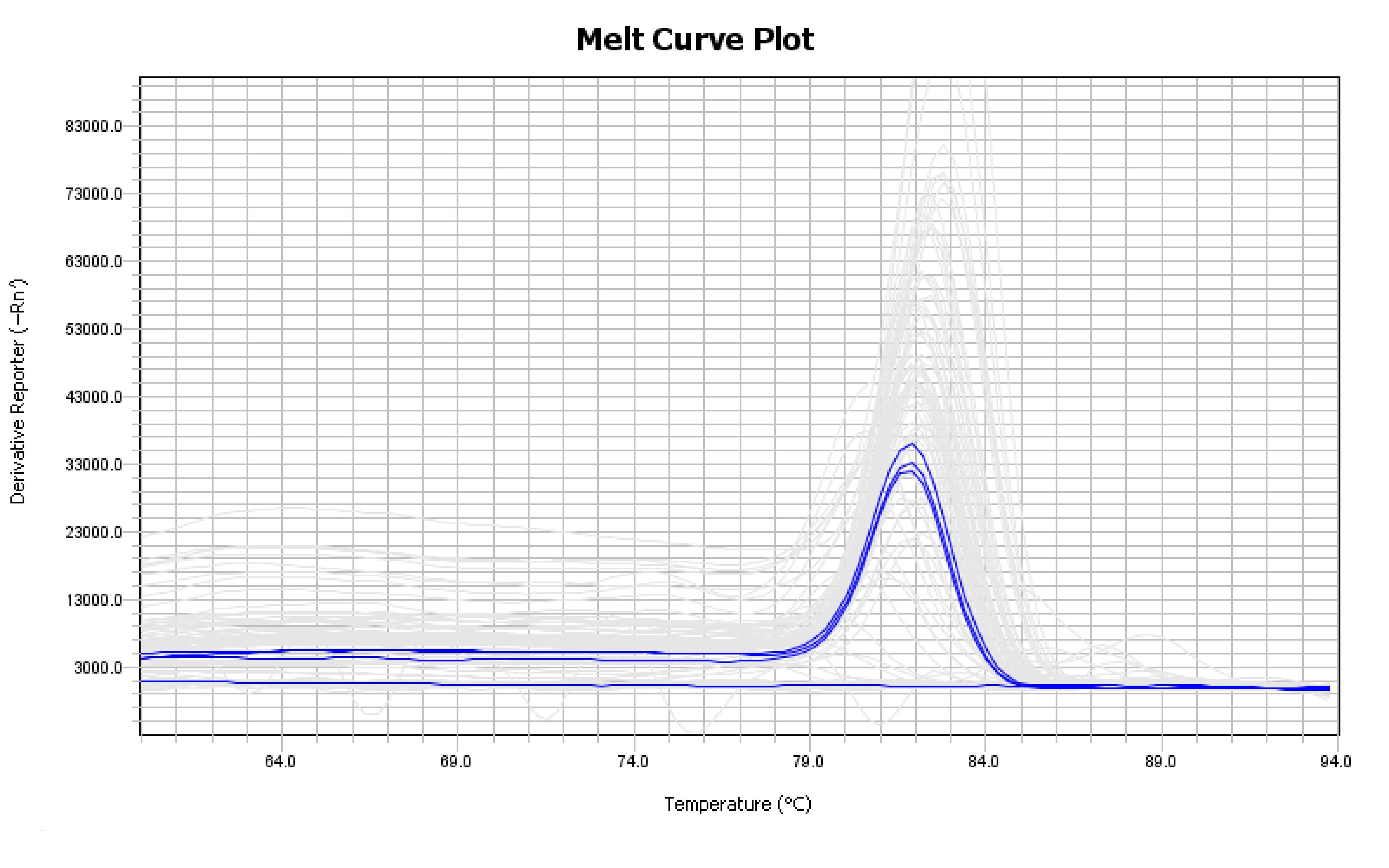

Supplement: Supplementary file 1 [file cimb-47-00536-s001.zip › Figure S1/K.jpg]

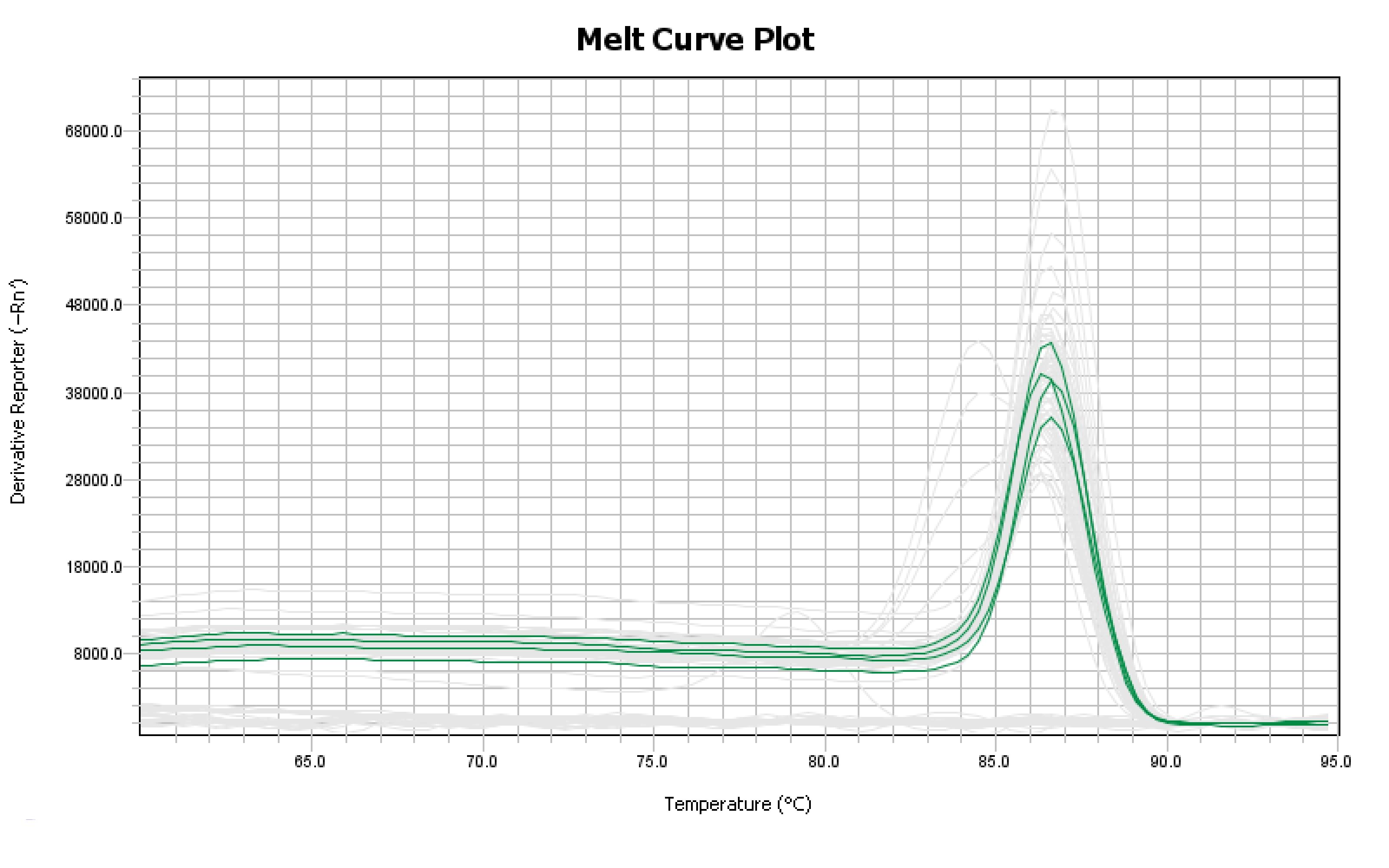

Supplement: Supplementary file 1 [file cimb-47-00536-s001.zip › Figure S1/L.jpg]
